# Retrieval Integrity Verification and Multi-System Data Interoperability Mechanism of a Blockchain Oracle for Smart Healthcare with Internet of Things (IoT) Integration

**DOI:** 10.3390/s24237487

**Published:** 2024-11-24

**Authors:** Ziyuan Zhou, Long Chen, Yekang Zhao, Xinyi Yang, Zhaoyang Han, Zheng He

**Affiliations:** 1 School of Computer Science, School of Cyber Science and Engineering, Engineering Research Center of Digital Forensics, Ministry of Education, Nanjing University of Information Science and Technology, Nanjing 210044, China; 202212490378@nuist.edu.cn (Z.Z.);; 2 School of Software, Shandong University, Jinan 250100, China; 3 ZHONGNENG Integrated Smart Energy Technology Co., Ltd., Beijing 100013, China

**Keywords:** Internet of Things, Blockchain Oracle, smart healthcare, retrieval integrity verification, cuckoo filter

## Abstract

The proliferation of Internet of Things (IoT) technology has significantly enhanced smart healthcare systems, enabling the collection and processing of vast healthcare datasets such as electronic medical records (EMRs) and remote health monitoring (RHM) data. However, this rapid expansion has also introduced critical challenges related to data security, privacy, and system reliability. To address these challenges, we propose a retrieval integrity verification and multi-system data interoperability mechanism for a Blockchain Oracle in smart healthcare with IoT Integration (RIVMD-BO). The mechanism uses the cuckoo filter technology to effectively reduce the computational complexity and ensures the authenticity and integrity of data transmission and use through data retrieval integrity verification. The experimental results and security analysis show that the proposed method can improve system performance while ensuring security.

## 1. Introduction

The rapid development of Internet of Things (IoT) technology has greatly promoted smart medical systems, enabling them to efficiently collect and process medical data, such as electronic medical records (EMRs) and remote health monitoring (RHM) data [1]. These data are crucial in supporting medical decision-making and patient management [2]. However, the widespread use of IoT devices also brings challenges in data security and privacy protection [3,4].

Smart healthcare systems increasingly rely on the integration of large amounts of external medical data to support more comprehensive and accurate diagnosis and treatment decisions [5,6]. At the same time, the rapid spread of IoT devices has expanded the sources and types of data, further increasing the need for efficient data integration [7]. External data face an increased risk of tampering, loss, or corruption during transmission, which may lead to inaccurate information entering the system. This not only affects the reliability of smart contract execution but also brings potential risks of diagnostic errors and ineffective treatment plans, thereby endangering patient safety and trust in the healthcare system [8]. Existing Blockchain Oracles, as a bridge between on-chain smart contracts and off-chain data sources, can cross the data barriers on and off the chain but still have limitations in ensuring data authenticity and integrity [9]. Therefore, there is an urgent need for an efficient verification mechanism to ensure the integrity and reliability of external data throughout the retrieval and integration process [10].

In addition, the heterogeneity of medical data systems and the diversity of data generated by IoT devices make it more complex to achieve seamless interoperability of multi-system data [11]. There are significant differences in the format, structure, and quality of data from different platforms such as EMR and RHM, which increases the difficulty of integrating and utilizing medical data and may lead to information fragmentation, affecting comprehensive patient care and scientific medical decision-making [12]. Therefore, there is an urgent need for an efficient retrieval integrity verification mechanism to ensure multi-system data interoperability and promote accurate and reliable data exchange between multiple systems, thereby establishing a cohesive healthcare ecosystem [13,14].

Finally, as the amount of external data continues to increase, it is difficult for existing verification methods to meet the needs of real-time medical decision-making in terms of computational efficiency and speed [15]. Many traditional verification methods have high computational complexity and slow processing speed, making it difficult to support the needs of real-time decision-making in intelligent medical systems [16,17]. Therefore, there is an urgent need for an efficient retrieval integrity verification technology to reduce computational costs and increase verification speed [18].

To address the above challenges, this paper proposes a smart medical Blockchain Oracle retrieval integrity verification and multi-system data interoperability mechanism (RIVMD-BO) suitable for the Internet of Things environment. The mechanism uses retrieval integrity verification technology to achieve the real-time verification of external medical data, ensuring the integrity and authenticity of data during cross-system transmission and use. In addition, the computational complexity of the verification process is reduced by introducing the cuckoo filter technology, thereby improving verification efficiency. Experimental results and security analysis show that the mechanism proposed in this paper can not only improve system performance but also effectively ensures the security of patient data in smart medical systems.

The main contributions of this paper are as follows:

(1) Aiming at the authenticity and integrity issues of external data in the IoT smart medical system, the RIVMD-BO mechanism is proposed, which provides effective support for achieving secure and reliable data interoperability.
(2) The verification process is optimized through the cuckoo filter, which significantly reduces the computational complexity and is suitable for the efficient cross-system transmission of medical data.
(3) Through comprehensive security analysis and performance evaluation, the effectiveness of the mechanism and its potential for application in intelligent medical systems are verified.

This paper is organized as follows: Section 2 reviews related work and the current status of medical information security and Blockchain Oracles. Section 3 and Section 4 introduce the design and security analysis of the RIVMD-BO mechanism, respectively. Section 5 introduces the experimental setup and analyzes the results. The effectiveness of the proposed mechanism is verified through experimental data, and its advantages and limitations in practical applications are discussed. Finally, the main contributions of this paper are summarized, and directions for future research are proposed.

## 2. Related Works

### 2.1. Traditional Healthcare Information System

Traditional healthcare information systems, such as EHR, Hospital Information Systems (HISs), and Laboratory Information Management Systems (LIMSs), have long relied on centralized databases for managing data [19]. Chenthara et al. [20] identified the primary challenges in securing electronic medical records, emphasizing that the integrity and reliability of medical data during sharing are paramount to prevent tampering. They also highlighted the importance of safeguarding security and confidentiality to prevent data breaches and outlined specific requirements for ensuring the security and privacy of data-sharing platforms. Zhang et al. [21] explored the security needs of electronic medical record systems in cloud computing environments and proposed strategies for securing medical data in cloud storage. Yang et al. [22] introduced a searchable encryption scheme, where cryptographic techniques are employed to protect the security of cloud-stored data. Xhafa et al. [23] developed an attribute-based electronic record system leveraging cloud computing technology, with a focus on protecting patient privacy. Sahi et al. [24] proposed a strategy for addressing potential storage security and privacy issues in cloud-based healthcare systems, suggesting the use of security measures, disaster recovery plans, and privacy protection techniques as effective methods to ensure the security and integrity of medical data.

Most studies focus on privacy in electronic medical record systems, but the centralized nature of cloud storage poses risks like tampering, data loss, and unauthorized access [25,26].

### 2.2. Blockchain-Based Healthcare Systems

While these traditional healthcare information systems have enhanced data management efficiency to some degree, they still encounter challenges like data silos, obstacles to information sharing, and the risk of data breaches. With the emergence of blockchain technology, its decentralized nature and integration with cryptographic techniques have increasingly found applications in medical information systems.

Zou et al. [27] proposed a blockchain medical data sharing and privacy protection system called SPChain, focusing on solving the problems of inefficient data retrieval and privacy risk in blockchain e-health systems. By designing special key blocks and microblocks, the system achieves efficient data sharing while protecting privacy and incentivizes the participation of healthcare providers through a reputation system. Liu et al. [28] proposed a blockchain-based Multi-Keyword Inner-Product Searchable Encryption scheme (MK-IPSE) aimed at enhancing the privacy protection and retrieval efficiency of EHR. Gao et al. [29] proposed a blockchain- and cloud–edge-computing-based electronic medical record-sharing scheme, which solves the problem of computational burden on resource-constrained devices while guaranteeing the fairness of data access. Through smart contracts and consistency algorithms, the scheme improves system efficiency while ensuring the integrity and security of medical data. Madine et al. [30] designed a personal health record management system based on the Ethereum blockchain, which empowers patients to control their own data.

In addition, Blockchain Oracles, as important intermediaries for the interaction between blockchain systems and external data, play an important role in solving the credibility problem in medical data sharing. Chen et al. [31] proposed a blockchain-based medical data-sharing mechanism to achieve the decentralized management of medical data through attribute-based access control and privacy protection. The scheme adopts the Hyperledger Fabric platform and uses a chain code to implement attribute access control so that only users with corresponding permissions can access medical data, enhancing the security and privacy of the data. In addition, K-anonymity and searchable encryption ensure that data do not leak privacy during sharing, and performance experiments show that the scheme has good effects in terms of scalability and security. At the same time, the application of Blockchain Oracles has also been explored in the credibility of cloud services. Zhou et al. [32] proposed a blockchain witness model, which introduces the role of “witness” and uses game theory and smart contract technology to detect and report service defaults. The model designs an incentive mechanism to ensure the credibility of witnesses and avoids witness bias or collusion through random algorithms, further improving the reliability of the system. Experiments show that the scheme has good application prospects in terms of performance and credibility.

## 3. Proposed RIVMD-BO

### 3.1. System Model

In the smart healthcare scenario, the Blockchain Oracle model involves four main entities: the healthcare blockchain network, the Blockchain Oracle, the healthcare external data source, and the healthcare user.

Medical user: Medical users, such as institutions or patients, create encrypted data tags for verifying data integrity before uploading medical records to external data sources, thus ensuring security while reducing storage and query costs.

External healthcare data sources: External healthcare data sources, such as cloud platforms or third-party services, store data and tags uploaded by healthcare users. Although usually reliable, these sources may still pose risks of data deletion or tampering.

Blockchain Oracle: The Blockchain Oracle acts as an intermediary, generating retrieval requests and verifying data integrity proofs to ensure accuracy. Based on verification outcomes, it adjusts trust scores for data sources, enhancing the reliability of future data retrievals.

Medical blockchain network: Medical blockchain networks, comprising healthcare entities like hospitals and insurers, use oracles to request and validate external data. Once verified, the data are used for applications such as clinical support and patient management.

**Figure 1:**
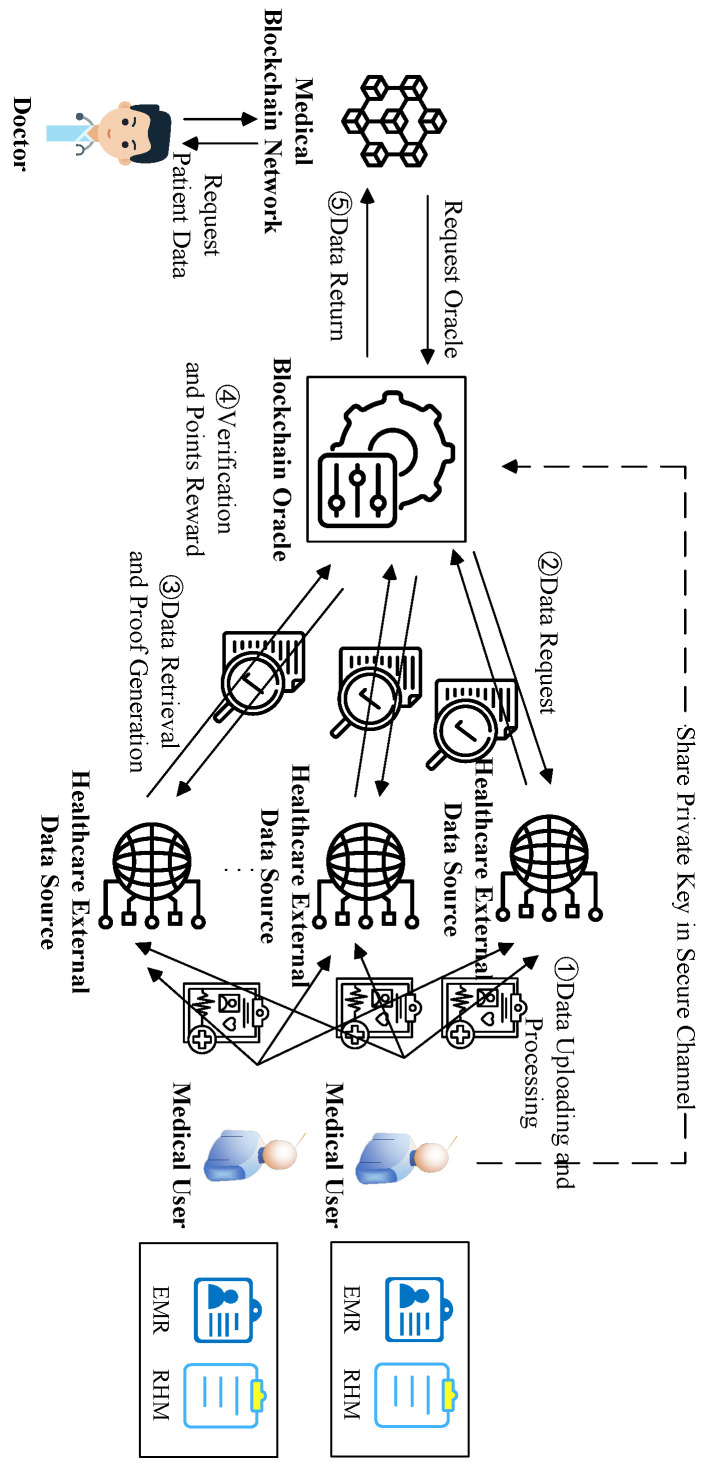
System model of RIVMD-BO.

To ensure secure data transmission and precise application within the smart healthcare system, this study develops a system model for data retrieval integrity verification using a Blockchain Oracle and a multi-system data interoperability mechanism. The system model is illustrated in Figure 1, and the detailed process for data retrieval and verification in practical scenarios is outlined below.

(1) Data Uploading and Processing
  Medical users process health check data such as EMR and RHM data into multiple data blocks and generate corresponding data labels for each data block. After encrypted processing, the data and labels are uploaded to a medical external data source through a secure channel. In addition, healthcare users share private keys for authentication with the smart healthcare Blockchain Oracle through the same secure channel. This process is designed to ensure the security of sensitive patient information and provide a solid foundation for subsequent data retrieval and validation.
(2) Data Request
  When doctors require access to specific medical data, they begin by submitting a data request to the medical blockchain network. Upon receiving this request, the network forwards a call request, including the identification details of the needed medical data, to the smart medical Blockchain Oracle. The oracle then formulates a retrieval request directed at an external medical data source, selecting the most reliable source based on the latest trust score to reduce the likelihood of data tampering or loss.
(3) Data Retrieval and Proof Generation
  After receiving the retrieval request, the medical external data source parses the data identification information in the request and begins to perform data retrieval operations to locate and retrieve the corresponding data blocks. Concurrently, it generates integrity verification certificates for these data blocks to confirm that the data are not tampered with and remain intact. The retrieved data and their integrity verification certificates are then sent to the smart healthcare Blockchain Oracle.
(4) Verification and Point Rewards
  The smart medical Blockchain Oracle performs data integrity validation immediately after receiving the retrieval results and validation proofs from the medical external data sources. The oracle rewards or penalizes the medical external data source based on the verification results and updates its trust points. This trust-based mechanism helps to build a reliable medical data ecosystem and guarantee the security and accuracy of subsequent data retrieval.
(5) Data Return
  The verified data are returned to the medical blockchain network by the smart medical Blockchain Oracle, and ultimately, the real medical data, which have been verified for retrieval integrity, are accessed by doctors through the blockchain network. These data provide strong support for clinical decision-making, treatment plan development, and patient management, ensuring the accuracy and reliability of medical services.

### 3.2. Security Model

In our work, the external data source is an incompletely trustworthy entity; i.e., it follows the “honest but curious” principle. During each ciphertext search execution, the external data source obtains and records as much information as possible about the encrypted document and index keywords and performs as much computation as possible to try to guess the plaintext information. It is important to note that the user and the blockchain predicator are like entities, i.e., believable entities. And in this work, we mainly consider that an external data source can continuously select keyword trapdoors in the search history to restore the document index; the process is called Indistinguishability under Chosen Keyword Attack (IND-CKA). The security model of this scheme against IND-CKA is given below. The security model is a polynomial time security game involving the adversary A and challenger C. The complete description is as follows.

(1) *Setup:* Challenger C inputs a security parameter $1^{\lambda }$ and runs the $KeyGen\left(1^{\lambda }\right)$ algorithm, which sends the generated system parameter params as well as the public key pk to the adversary A. Challenger C keeps the private key sk.
(2) *QueryPhase1:* Adversary A adaptively selects a series of keyword sets $\left\{Q_{1},\cdots ,Q_{h}\right\}$ at random and sends them one by one to challenger C. Challenger C executes the Trapdoor (params,sk,w) algorithm to generate the trapdoor $T_{h}$ corresponding to each keyword set and sends it back to adversary A.
(3) *Challenge:* Adversary A selects a keyword set $Q^{*}$ and sends it to challenger C. Challenger C selects a random keyword set $R^{*}$. C sets $W_{0}=Q^{*}$ and $W_{1}=R^{*}$ and selects 1 random bit β∈{0,1}; at the same time, to run the $BuildIndex\left(W_{\beta },params,pk\right)$ algorithm, it generates the corresponding index $S_{\beta }$ of the keyword set $W_{\beta }$. After that, challenger C sends the ternary $\left(W_{0},W_{1},S_{\beta }\right)$ to adversary A.
(4) *QueryPhase2:* Adversary A then additionally adaptively selects a series of keyword sets $Q_{h+1},\cdots ,Q_{\lambda }$, which cannot include $W_{0}$ or $W_{1}$ returned from the challenge phase. It sends these keyword sets in turn to challenger C, who runs the Trapdoor $\left(params,sk,w\right)$ algorithm to generate the corresponding trapdoor $T_{\lambda }$ for each keyword set and send it back to A. The number of queries by adversary A is *t* in probabilistic polynomial time.
(5) *Guess:* Adversary A needs to output either $\beta^{\prime }=0$ or $\beta^{\prime }=1$ as a judgment on the random value chosen by C. The adversary A is required to output either $\beta^{\prime }=0$ or $\beta^{\prime }=1$ as a judgment. If $\beta =\beta^{\prime }$, then A wins the game, and if not, A loses the game.

For any polynomial time adversary A, its advantage of winning this security game is denoted as ${\mathrm{Adv}}_{A}\left(1^{\lambda }\right)=\left|\mathrm{Pr}\left(\beta =\beta^{\prime }\right)-1/2\right|$. Conditional on the security parameter $1^{\lambda }$, the scheme is said to be effective against IND-CKA if the advantage of adversary A is a negligible function.

**Definition 1.**  *Let g be an arbitrary generator in a cyclic group G of order prime P, and le a,b be random elements in the group $Z_{q}$ of positive integers. Given $\left(g,g^{a},g^{b}\right)$, output $g^{ab}$. If it is computationally infeasible to compute $g^{ab}$ using the given tuple $\left(g,g^{a},g^{b}\right)$, then the Computational Diffie–Hellman (CDH) assumption in G holds. Suppose that an attack algorithm is trying to solve the CDH problem in group G. The advantage of its successful solution is denoted as ${\mathrm{Adv}}_{A}^{\mathrm{CDH}}=Pr\left[A\left(g,g^{a},g^{b}\right)=g^{ab}\right]$.*

In the case where the parameters in attack algorithm are all chosen randomly, attack algorithm runs for at most time *t* with an advantage of at least ε for a successful solution. The (t,ε)-CDH problem is said to hold in group *G* if there does not exist any time-t algorithm that has an advantage of at least ε for solving the CDH problem.

### 3.3. Construction of RIVMD-BO

**Figure 2:**
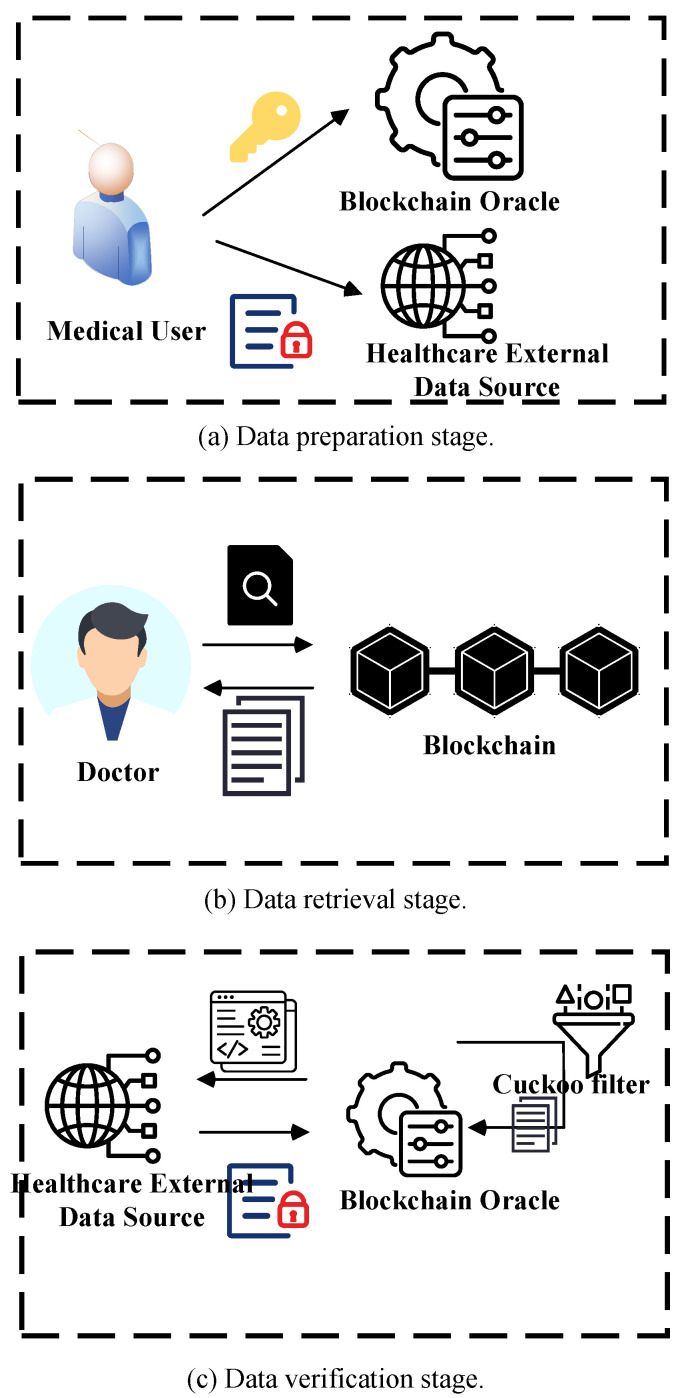
Main steps of the mechanism.

In this section, we specify the construction of the RIVMD-BO mechanism, which consists of the following phases: (1) system setup phase; (2) data preparation phase; (3) data retrieval phase; and (4) data verification phase. In addition, the main steps of the Blockchain Oracle data retrieval integrity verification method are shown in Figure 2.

Suppose $G_{1}$, $G_{2}$ and $G_{T}$ are three multiplicative cyclic groups of prime *q*, where $g_{1}$ is the generating element of $G_{1}$, $g_{2}$ is the generating element of $G_{2}$, and $e:\;G_{1}\;\times \;G_{2}\;\to \;G_{T}$ is a bilinear map. P: K × M ↦K is pseudo-randomized permutation, where K and M have the same length. $h:\;{\left\{0,\;1\right\}}^{*}\;\to \;G$ is the global hash function. $\Pi \;=\;\left(KeyGen,\;Enc,\;Dec\right)$ is an IND-CPA secure symmetric encryption scheme.

**Figure 3:**
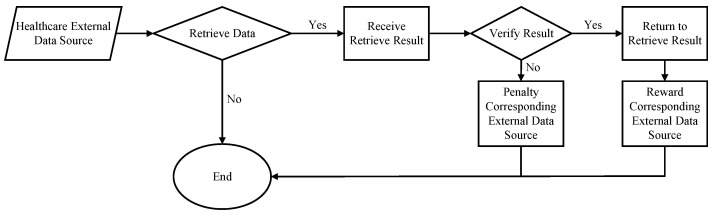
Trust points management workflow.

(1) System setup phase: The user initializes the system parameters. Input security parameters λ, and output system parameters $params\;=\;\{G_{1},\;G_{2},\;G_{T},\;e,\;g_{1},\;g_{2},\;h,\;P_{key}\}$, where $G_{1}$, $G_{2}$ and $G_{T}$ are the three multiplicative cyclic groups of prime *q*, *e* is the bilinear pairwise mapping, and $g_{1}$, $g_{2}$ are the generators of the groups $G_{1}$, $G_{2}$. *h* is the global hash function, and $P_{key}$ is a pseudo-randomized permutation controlled by key.
  The user chooses a random number $x\;\in \;Z_{P}^{*}$ as their private key sk and puts $\left(u,\;v\right)$ as the public key pk, where $u\;\leftarrow \;g_{1}^{x},\;v\;\leftarrow \;g_{2}^{x}$, to obtain the public–private key pair (sk, pk). Subsequently, the public key is made public and the private key is shared with the blockchain oracle.
(2) Data Preparation Phase: Assume that the data owner wants to upload a relational database $D\;=\;\left(A_{1},\;A_{2},\;\cdots ,\;A_{n}\right)$ to an external data source; for each data tuple to be uploaded, construct it as $r_{i}\;=\;\left(a_{i1},\;a_{i2},\;\cdots ,\;a_{in}\right)\left(i\;=\;1,\;2,\;\cdots \right)$, where $a_{ij}\;\in \;Z_{N}\left(j\;=\;1,\;2,\;\cdots ,\;n\right)$. Note that each attribute $A_{j}$ in the database discussed in this paper is the keyword entered during the search operation.
  Cuckoo filter is an efficient data structure used to determine whether data exist in a set. It uses two hash functions to calculate two possible storage locations for each data item and can rearrange existing data items when conflicts occur, thereby achieving efficient storage and search.
  Create the initial filter structure: Each cuckoo filter consists of two hash buckets, each of which can hold multiple fingerprints. The size and number of hash buckets are set in advance to ensure the success rate of data insertion and reduce conflicts.
  Calculate data item positions and generate fingerprints: For each data item, we generate two bucket positions through a hash function. First, the hash function is applied to generate the first position, and then the second position is generated through an XOR operation so that two positions are obtained for insertion selection. In addition, in order to save storage space and ensure the uniqueness of verification, a fixed-length fingerprint is generated for the data item. The fingerprint is a hashed simplified identifier that can reduce storage requirements while ensuring data accuracy.
  Insert data items and resolve conflicts: When inserting data, the fingerprint is first stored in the first available bucket position. If both bucket positions are occupied, the cuckoo filter performs a kick-out operation; that is, it randomly replaces the existing fingerprint, makes room for the new fingerprint, and finds a new position for the replaced fingerprint. This ensures a high insertion success rate and can handle a large number of data items even under high load. In order to avoid loops that may be caused by insertion conflicts, the cuckoo filter is designed with a limited retry mechanism. Once the limit is exceeded, the filter capacity is expanded to ensure that the insertion process proceeds smoothly.
  For any value $a_{ij}$ in tuple $r_{i}$, compute $k_{i}^{\prime }\;=\;P_{k_{0}}\left(i\right)$ and encrypt $a_{ij}$ as $c_{ij}\;=\;Enc_{k_{i}}\left(f_{ij}\;\|\;a_{ij}\right)$. For any value $a_{ij}$ in tuple $r_{i}$, compute $s_{ij}\;=\;P_{k_{2}}\left(a_{ij}\right)$, $k_{s_{ij}}\;=\;P_{k_{1}}\left(a_{ij}\right)$, and the corresponding labeled attribute value $a_{ij}$ as $t_{ij}\;=\;Enc_{k_{s_{ij}}}\left(s_{ij}\right)$. For each tuple $r_{i}\;=\;\left(a_{i1},\;a_{i2},\;\cdots ,\;a_{in}\right)\left(i\;=\;1,\;2,\;\cdots \right)$, record its ciphertext tuple as $r_{i}^{E}\;=\;\{\left(t_{i1},\;c_{i1},\;A_{1}\right),\;$$\left(t_{i2},\;c_{i2},\;A_{2}\right),\;\cdots ,\;\left(t_{in},\;c_{in},\;A_{n}\right)\}$ and generate the signature $\sigma_{i}\;\leftarrow \;{\left(h\left(i\right)g_{1}^{r_{i}^{E}}\right)}^{x}$.
  The user then needs to construct the Merkle hash accumulator $ACC\;=\;{\left\{ACC_{j}\right\}}_{1\leq j\leq n}$ with the signature set $\Phi \;=\;\{\sigma_{i}\},\;1\;\leq \;i\;\leq \;n$ as the leaf node. For each attribute $A_{j}\left(j\;=\;1,\;2,\;\ldots ,\;n\right)$, construct the cuckoo filter $CF_{j}$; first create an empty hash table, and then construct the corresponding two buckets for each attribute $A_{j}$, and then, according to Equation (1)’s insertion algorithm, to compute the position of all the nodes, construct the cuckoo filter $CF\;=\;{\left\{CF_{j}\right\}}_{1\leq j\leq n}$.
  (1) $$\begin{array}{c} i_{1}\;=\;h\left(x\right),\;i_{2}\;=\;i_{1}\;⊕\;h\left(fp\right),\;fp\;=\;Fingerprint\left(x\right) \end{array}$$
  where $i_{1}$ and $i_{2}$ are the locations of the two buckets, *h* is the hash function that computes the location of the bucket, and Fingerprint is the hash function that computes the fingerprint.
  Finally, the ciphertext tuple $\left(r_{i}^{E},\sigma_{i}\right)$ and the metadata SC consisting of the signature $\sigma_{ij}$, the cuckoo filter CF, and the Merkle accumulator ACC are sent to the external data source.
(3) Data retrieval phase: the Blockchain Oracle submits a retrieval request to an external data source, assuming that the blockchain network wants to search for all tuples whose value in attribute $A_{j}$ is $a_{q}$ (denoted as $A_{j}\;=\;a_{q}$). The user generates a retrieval request *T* based on the keyword w that it wishes to retrieve and the key *K* as input, where $T\;=\;\left(q,\;k_{q},\;A_{j}\right)\;=\;\left(P_{k_{2}}\left(a_{q}\right),\;P_{k_{1}}\left(a_{q}\right),\;A_{j}\right)$, and then sends *T* sent to the external data source.
  After receiving the retrieval request *T*, the external data source checks the label $t_{ij}\left(i\;=\;1,\;2,\;\cdots \right)$ corresponding to the attribute $A_{j}$ element by element to verify whether $Dec_{k_{q}}\left(t_{ij}\right)\;=\;q\left(i\;=\;1,\;2,\;\cdots \right)$ holds. All tuples of ciphertexts for which $t_{ij}$ satisfies the condition are $\left\{r_{i_{1}}^{E},\;r_{i_{2}}^{E},\;\cdots ,\;r_{i_{l}}^{E}\right\}$. Generate the corresponding aggregated signature $\sigma \;\leftarrow \;\prod_{i=1}^{l}\;\sigma_{i}^{\alpha_{i}}$, where $\alpha_{i}$ is a random element $\alpha_{i}\;\leftarrow \;R$. The external data source generates a proof $\pi \;=\;\left(\sigma ,\;\mu \right)$ where $\mu \;=\;\sum_{i=1}^{l}\;r^{E}\alpha_{i}$. Finally, the corresponding result and proof $\left(r_{i_{1}}^{E},\;r_{i_{2}}^{E},\;\ldots ,\;r_{i_{l}}^{E},\;\pi \right)$ are sent to the blockchain oracle.
(4) Data verification phase: the Blockchain Oracle performs integrity verification of the results received from the external data sources by verifying the completeness and correctness of the checking results through the cuckoo filter.
  Data retrieval integrity verification aims to ensure the accuracy of data during transmission and use. In the verification phase, the cuckoo filter is used to quickly check whether the data have been tampered with and to achieve efficient authenticity verification by verifying whether each data item matches the hash position in the filter.
  Lookup operation and fingerprint comparison: When receiving the data item to be verified, the oracle will calculate the two positions of the item through the hash function and generate the corresponding fingerprint. Then, it will check whether the two bucket positions of the filter contain the fingerprint. If a matching fingerprint is found, it means that the data item has been successfully recorded when it was uploaded and meets the integrity requirements.
  Measures for verification failure: If no fingerprint match is found in either position, the system determines that the data item may be lost or tampered with. At this time, the oracle will record the abnormal situation and issue an alarm and consider whether the data item fails to pass the verification for other reasons, so as to take further processing measures. This design ensures the consistent verification of data items and improves the fault tolerance of the oracle to abnormal data.
  Firstly, the correctness of the result is verified by verifying the validity of Equation (2). Then, after determining the validity of the signatures, the oracle performs a cuckoo filter lookup operation based on Equation (3) to check whether all the signatures exist in the cuckoo filter. If all signatures exist in the cuckoo filter, the retrieval integrity verification passes; otherwise, the retrieved data are compromised.
  (2) $$\begin{array}{cc} e\left(\sigma ,g_{2}\right) & \;=\;e\left(\prod_{i=1}^{l}h\left(i\right)g_{1}^{\mu },v\right) \end{array}$$
  (3) $$\begin{array}{c} \mathrm{Query}(x)\;=\;\left(\mathrm{Filter}\left[i_{1}\right]\;=\;fp\right)\;\vee \;\left(\mathrm{Filter}\left[i_{2}\right]\;=\;fp\right) \end{array}$$
  As illustrated in Figure 3, once the Blockchain Oracle receives the retrieval results and verification proofs from the external data source, it begins the process of data integrity verification. Depending on the outcome of this verification, the Blockchain Oracle uses Algorithm 1 to either reward or penalize the external data source, adjusting the associated trust points accordingly. The algorithm dynamically adjusts the trust points by considering the current service quality, historical performance, and behavioral stability of the data source and combines them with a delayed punishment mechanism to ensure the security and accuracy of data transmission in the system.
  First, if the data source provides correct data, the trust integral ts will be positively updated according to the preset speed factor α. Conversely, if the data source provides incorrect or malicious data, ts will be negatively adjusted according to the same α. Meanwhile, the latency delay will be updated according to the verification results, and correct data sources will decrease the delay according to the delayed update speed factor β, while incorrect data sources will increase delay. In order to further adjust the trust scores, the algorithm introduces the historical quality of service weight γ, the historical quality of service *h*, and the behavioral fluctuation factor ϵ. *h* affects the the magnitude of the adjustment of the trust score, and ϵ is used to penalize data sources whose historical performance differs significantly from the current performance, thus ensuring that the stability of the data source is reflected in the trust score. Finally, the algorithm sets a boundary condition for the trust integral to ensure that it is always in the range [0,100]. If the trust integral falls below a specific threshold θ, the delay penalty is further increased to prevent unreliable data sources from continuing to occupy system resources. The trust integral and delay after these adjustments are used as the final output to guide the subsequent data retrieval and verification process.
**Algorithm 1** Trust score update
1:** Input:** verify(), ts, delay, α, β, γ, h, ϵ, θ2:
** Output:**  ts, delay

3:** if** verify() = 1 **then**4:        $ts_{new}\;=\;ts\;+\;\alpha \;\cdot \;\left(1\;-\;\frac{ts}{100}\right)$5:        delay = delay · (1 − β)6:
** else**
7:        $ts_{new}\;=\;ts\;-\;\alpha \;\cdot \;\left(1\;-\;\frac{ts}{100}\right)$8:        $delay\;=\;delay\;+\;\left(\frac{100\;-\;ts}{100}\right)\;\cdot \;\beta$9:
** end if**
10: $ts_{adjusted}\;=\;ts_{new}\;\cdot \;\left(1\;+\;\gamma \;\cdot \;\frac{h}{100}\right)\;\cdot \;\left(1\;-\;\epsilon \;\cdot \;\frac{\left|h\;-\;ts\right|}{100}\right)$11: $ts_{final}\;=\;\max\left(0,\;\min\left(100,\;ts_{adjusted}\right)\right)$12:
** if** $ts_{final}\;\leq \;\theta$ **then**
13:        $delay\;=\;delay\;+\;\frac{\theta \;-\;ts_{final}}{\theta }$14:
** end if**
15:
** Return:** ts, delay

  In addition, this scheme designs a dynamic trust score protection algorithm to ensure that the system’s trust score can remain robust and reliable even if the oracle is attacked or tampered with. In Algorithm 2, first, the data source scoring of each round is monitored, the trust score increment Δts and delay increment Δdelay of the current round are calculated, and the historical average increment $\Delta ts_{avg}$ and $\Delta delay_{avg}$ are calculated based on the rolling window. Then, the set thresholds $\sigma_{ts}$ and $\sigma_{delay}$ are used to determine whether there is an anomaly. When an anomaly is detected, the current trust score and delay are dynamically adjusted according to the factor ν to prevent the abnormal change from having too much impact on the overall system. Finally, the updated trust score and delay value will be used for the next round of scoring and protection evaluation to form a dynamic protection cycle.
**Algorithm 2** Dynamic trust score protection
1:** Input:** $ts,\;delay,\;\Delta ts,\;\Delta delay,\;\sigma_{ts},\;\sigma_{delay},\;\lambda ,\;\nu$2:** Output:** $ts_{protected},\;delay_{protected}$3:$\;\Delta ts_{avg}\;\leftarrow \;\frac{1}{\lambda }\sum_{i=1}^{\lambda }\;\Delta ts\left[i\right]$                  ▹ Average recent change in trust score4:$\;\Delta delay_{avg}\;\leftarrow \;\frac{1}{\lambda }\sum_{i=1}^{\lambda }\;\Delta delay\left[i\right]$                ▹ Average recent change in delay5:
** if** $\;|\Delta ts\;-\;\Delta ts_{avg}|\;>\;\sigma_{ts\;}$ **then**
6:        $ts_{protected}\;=\;ts\;-\;\nu \;\cdot \;\left(\Delta ts\;-\;\Delta ts_{avg}\right)$7:
** else**
8:        $ts_{protected}\;=\;ts$9:
** end if**
10:
** if** $\;|\Delta delay\;-\;\Delta delay_{avg}|\;>\;\sigma_{delay\;}$ **then**
11:        $delay_{protected}\;=\;delay\;+\;\nu \;\cdot \;\left(\Delta delay\;-\;\Delta delay_{avg}\right)$12:
** else**
13:        $delay_{protected}\;=\;delay$14:
** end if**
15:** Return:** $ts_{protected},\;delay_{protected}$

## 4. Security Analysis

From the definition of security, it is clear that the security of this scheme is based on the CDH problem. Therefore, in the security analysis, it will be shown that the scheme is IND-CKA-secure under the CDH assumption of the random oracle model.

**Lemma 1.**  *In the random oracle model, let adversary A query the keyword set $W\;=\;\{w_{i}\}$ and construct a hash list $H_{\mathrm{list}}$. If a keyword $w_{i}$ is not in $H_{\mathrm{list}}$, the random oracle will return a unique new trapdoor and record it in $H_{\mathrm{list}}$.*

**Proof.**  In the random oracle model, when adversary A makes a query request, the random oracle will check whether the keyword $w_{i}$ already exists in $H_{\mathrm{list}}$. If not, the oracle will generate new random values $d_{i},e_{i}$ and record them in $H_{\mathrm{list}}$ to ensure that each keyword uniquely corresponds to a trapdoor. If the keyword $w_{i}$ already exists, the corresponding trapdoor is returned. This method ensures a one-to-one mapping relationship between keywords and trapdoors, effectively supporting the uniqueness of trapdoors in security analysis. □

**Lemma 2.**  *In the trapdoor query phase, if all keywords $w_{j}$ queried by adversary D satisfy ${\mathrm{coin}}_{j}\;=\;0$, adversary A can generate a random number s and successfully generate a trapdoor R; if there is a keyword $w_{j}$ that satisfies ${\mathrm{coin}}_{j}\;=\;1$, adversary A chooses to abstain from this query.*

**Proof.**  Adversary A decides whether to generate a valid trapdoor based on the *coin* value in the query result. If all query keywords satisfy coin = 0, adversary A generates a random number s and returns a valid trapdoor *R*; if there is any keyword with coin = 1, the adversary chooses to abstain. This mechanism ensures that the adversary only generates trapdoors under specific conditions, effectively supporting the subsequent challenge process. □

**Theorem 1.**  *In the random oracle model, if the group G satisfies the CDH assumption and there exists an adversary A running in polynomial time that can win the IND-CKA attack in the game specified in the security model with non-negligible probability ε, then the simulator B can solve the CDH problem in probabilistic polynomial time with a probability no less than $4kq_{t}\varepsilon$, where $q_{t}$ is the maximum number of trapdoor queries from adversary A to adversary B, $q_{h}$ is the maximum number of trapdoor queries from adversary A to a random oracle, k is the maximum number of keywords in a query request, ε is the advantage of adversary A in solving the CDH problem, and $t^{\prime }$ is the execution time of the algorithm.*

**Proof.**  Let A be an adversary in probabilistic polynomial time that performs an IND-CKA attack on this scheme. The hash function *h* in the system parameter params is modeled by the random oracle model. Now, another new polynomial time adversary B is set to utilize adversary A to solve the CDH problem; i.e., adversary A and adversary B jointly participate in the security game.

The proof process is divided into five parts: (1) the setup phase, (2) the initial query phase, (3) the challenge phase, (4) the subsequent query phase, and (5) the guess phase. The detailed steps of each part are as follows.

(1) *Setup:* Challenger C first gives the relevant parameters $\left\{G_{1},\;G_{2},\;G_{T},\;g_{1},\;g_{2}\right\}$ of the CDH problem to the group *G* in Definition 1 and sends them to the adversary B, where the parameters are defined as shown in Equation (4).

(4) $$\begin{array}{c} \nu_{1}\;=\;g_{1}^{a},\;\nu_{2}\;=\;g_{2}^{b},\;a,\;b,\;z\;\in \;Z_{p}^{*} \end{array}$$

Adversary B sets $g\;=\;g_{1},\;y\;=\;g_{2}$ and sets the private key sk = x to satisfy $g_{1}^{x}\;=\;g_{2}$. Additionally, B will secretly select a random number $\mu \;\in \;Z_{P}^{*}$ that will be used in the random oracle model and the challenge phase.

At the same time, B will pick a security parameter $1^{\lambda }$ and execute the $KeyGen\left(1^{\lambda }\right)$ algorithm to generate the system parameters $params\;=\;\{G_{1},\;G_{2},\;G_{T},\;e,\;g_{1},\;g_{2},\;h,\;P_{key}\}$, and it will send the params and the public key pk to A. Adversary A submits up to $q_{h}$ keyword queries to the random oracle, which responds to these queries by returning to A the trapdoor corresponding to the keyword. Adversary B stores a hash list $H_{list}$, as shown in Equation (5).

(5) $$\begin{array}{c} H_{list}:\;\left\{w_{i},\;coin_{i};\;h_{i},\;d_{i},\;f_{i},\;e_{i};\;p_{i}\right\} \end{array}$$

If a keyword $w_{i}$ has already been queried, then B returns Equation (6) to A.

(6) $$\begin{array}{c} h_{i}\;=\;H_{1}\left(w_{i}\right),\;f_{i}\;=\;H_{2}\left(w_{i}\right),\;p_{i}\;=\;H_{3}\left(w_{i}\right) \end{array}$$

If the keyword $w_{i}$ is not queried, B chooses a random value $p_{i}\;\in \;Z_{P}^{*}$ for $p_{i}$ and flips a random $coin_{i}\;\in \;\left\{0,\;1\right\}$. If this coin is set to 1, its probability is $1/kq_{t}$; if the coin is set to 0, its probability is $1\;-\;1/kq_{t}$. When the coin is set to 0, then B chooses two random numbers $d_{i},\;e_{i}\;\in \;Z_{P}^{*}$, so that $h_{i}\;=\;g_{1}^{d_{i}},\;f_{i}\;=\;g_{2}^{e_{i}}$; if the *coin* is set to 1, then B chooses a random number $d_{i}\;\in \;Z_{P}^{*}$, and performs the calculation in Equation (7).

(7) $$\begin{array}{c} e_{i}\;=\;d_{i}/\mu \end{array}$$

Finally, it returns the obtained $h_{i},\;f_{i},\;p_{i}$ to the adversary A and adds $\left\{w_{i},\;coin_{i};\;h_{i},\;d_{i},\;f_{i},\;e_{i};\;p_{i}\right\}$ to the list $H_{list}$.

(2) *QueryPhase1:* An adversary A adaptively queries a series of trapdoors for a collection of keywords. Let the set of keywords for a particular query be *Q* and the returned trapdoor be *T*. B obtains the corresponding tuples of the query keywords in the list $H_{list}$. If there is at least one $coin_{u}\;=\;1$, B chooses to abstain; if all of the $coin_{u}$ values are 0, B generates a random number $t\;\in \;Z_{P}^{*}$, and *t* will be updated in the next successful trapdoor query. Subsequently, B outputs the trapdoor shown in Equation (8).

(8) $$\begin{array}{c} T\;=\;\{T_{a},\;T_{b},\;T_{c},\;q_{1},\;\cdots ,\;q_{u}\} \end{array}$$

where, $T_{a},\;T_{b},\;T_{c}$ are shown in Equation (9).

(9) $$\begin{array}{c} T_{a}\;=\;g_{1}^{t},\;T_{b}\;=\;g_{1}^{t\left(\sum_{u=1}^{m}\;d_{u}\right)},\;T_{c}\;=\;g_{1}^{t\left(\sum_{u=1}^{m}\;e_{u}\right)},\;t\;\in \;Z_{p}^{*} \end{array}$$

Finally, B feeds the trapdoor *T* back to A.

(3) *Challenge:* Adversary A chooses a keyword set $Q^{*}$ and sends it to Adversary B. Adversary B additionally chooses a keyword set $R^{*}$. Keyword set $R^{*}$ and keyword set $Q^{*}$ cannot be present in the previous query of Adversary A. B sets $W_{0}\;=\;Q^{*}$ and $W_{1}\;=\;R^{*}$ and chooses a random bit β ∈ {0, 1} to obtain the set of keywords $W_{\beta }$ in Equation (10).

(10) $$\begin{array}{c} W_{\beta }\;=\;\left\{w_{\beta ,1},\;w_{\beta ,2},\;\cdots ,\;w_{\beta ,m}\right\} \end{array}$$

After that, B asks about all the keywords in $W_{\beta }$ one by one to the random oracle and returns the corresponding tuple of $H_{list}$; if there is no $coin_{\beta ,u}\;=\;1$, then B abstains. Instead, B generates the challenge ciphertext $S_{\beta }$ in Equation (11).

(11) $$\begin{array}{c} S_{\beta }\;=\;\left\{A,\;B,\;C_{\beta ,1},\;\cdots ,\;C_{\beta ,u}\right\} \end{array}$$

The elements in $S_{\beta }$ are defined as shown in Equation (12).

(12) $$\begin{array}{c} A\;=\;\nu_{1},\;B\;=\;\nu_{2}^{\eta },\;C_{\beta ,u}\;=\;\vec{L_{W_{\beta }}}\left(c_{\beta ,u}\right) \end{array}$$

If $coin_{\beta ,u}\;=\;0$, $c_{\beta ,u}$ performs the calculation shown in Equation (13).

(13) $$\begin{array}{cc} c_{\beta ,u} & \;=\;v_{1}^{d_{\beta ,u}}v_{2}^{e_{\beta ,u}\eta }\\ & \;=\;g_{1}^{ad_{\beta ,u}}g_{2}^{d_{\beta ,u}\eta }\\ & \;=\;h_{\beta ,u}^{a}f_{\beta ,u}^{\eta b} \end{array}$$

If $coin_{\beta ,u}=1$, $c_{\beta ,u}$ performs the calculation shown in Equation (14).

(14) $$\begin{array}{cc} c_{\beta ,u} & \;=\;v^{d_{\beta ,u}}\\ & \;=\;g^{abd_{\beta ,u}}\\ & \;=\;g^{ad_{\beta ,u}}{\left(g^{d_{\beta ,u}\beta }/\eta \right)}^{b\eta }\\ & \;=\;{\left(h_{\beta ,u}\right)}^{a}{\left(f_{\beta ,u}\right)}^{b\eta } \end{array}$$

Finally B sends the ternary $\left(W_{0},\;W_{1},\;S_{\beta }\right)$ to the adversary A.

(4) *QueryPhase2:* Adversary A continues with a series of trapdoor queries for a set of keywords that cannot be $W_{0}$ and $W_{1}$. B performs the same response as the pre-challenge query of QueryPhase1.

(5) *Guess:* Eventually, output the guess result $\beta^{\prime }\;=\;0$ or $\beta^{\prime }\;=\;1$. If $\beta \;=\;\beta^{\prime }$, the adversary B outputs the judgment result $v\;=\;g^{ab}$ for the CDH problem. Conversely, output the result v = z. □

The prerequisite for the adversary B to be successful is that they need to carry out the whole process of this security game completely; i.e., they cannot abstain in the trapdoor query phase and the challenge phase. Based on the fact that adversary A can make at most $q_{t}$ trapdoor queries and *k* is the maximum number of keywords in the query request, the probability that B does not abstain in the trapdoor query phase and the challenge phase is shown in Equation (15) and Equation (16), respectively.

(15) $$\begin{array}{c} \mathrm{Pr}\;\left(B\;pass\;Queryphase\;1\;and\;2\right)\;=\;{\left(1\;-\;1/kq_{t}\right)}^{kq_{t}} \end{array}$$

(16) $$\begin{array}{c} Pr\;\left(B\;pass\;Challenge\right)\;=\;\left[1\;-\;{\left(1\;-\;1/kq_{t}\right)}^{k}\right] \end{array}$$

From the above, $kq_{t}\;\in \;\left[2,+\infty \right)$, ${\left(1\;-\;1/kq_{t}\right)}^{kq_{t}}$ is a monotonically increasing function in the interval with a minimum value of ${\left(1\;-\;1/kq_{t}\right)}^{kq_{t}}\;\geq \;1/4$, and $\left[1\;-\;{\left(1\;-\;1/kq_{t}\right)}^{k}\right]\;\geq \;\left[1\;-\;\left(1\;-\;1/kq_{t}\right)\right]\;=\;1/kq_{t}$, so the probability of B completing the game is shown in Equation (17).

(17) $$\begin{array}{cc} & \mathrm{Pr}\;\left(B\;pass\;Game\right)\;=\;{\left(1\;-\;1/kq_{t}\right)}^{kq_{t}}\\ & \left[1\;-\;{\left(1\;-\;1/kq_{t}\right)}^{k}\right]\;\geq \;1/4kq_{t} \end{array}$$

From the conditions in Theorem 1, the advantage of successful guessing by Adversary A is ε non-negligible, which leads to the conclusion in Equation (19) that the advantage of Adversary B in obtaining victory is $\varepsilon /4kq_{t}$.

(18) $$\begin{array}{c} \mathrm{Ad}v_{B}^{\mathrm{CDH}}\left(1^{\lambda }\right)\;\geq \;1/4kq_{t}\left|\mathrm{Pr}\left(\beta \;=\;\beta^{\prime }\right)\;-\;1/2\right|\;=\;\varepsilon /4kq_{t} \end{array}$$

Therefore, the advantage of adversary B to solve the CDH problem is not negligible. It can be proved that this scheme is $\left(t^{\prime },\;q_{t},\;q_{h},\;4kq_{t}\varepsilon \right)$-safe against the IND-CKA attack under the random oracle model.

**Security analysis of trust score dynamic protection algorithm**

In this solution, the dynamic protection mechanism of the data source trust score is designed to defend against possible manipulation attempts by attackers. We use a series of mathematical models and adaptive threshold judgment methods to ensure that even if the oracle is compromised in a certain round, the adjustment of the trust score will not be significantly affected. To this end, the dynamic trust score protection mechanism uses the following analysis:

In round *t*, the changes in trust score and delay are calculated as

(19) $$\begin{array}{c} \Delta ts_{avg}\;=\;\frac{1}{\lambda }\sum_{i=t-\lambda +1}^{t}\Delta ts\left[i\right],\;\;\;\;\Delta delay_{avg}\;=\;\frac{1}{\lambda }\sum_{i=t-\lambda +1}^{t}\Delta delay\left[i\right] \end{array}$$

These averages measure normal trends in trust scores and latency. We compare dynamically detected deviations in trust scores and latencies against thresholds to ensure that the system can quickly isolate anomalous impacts when under attack. The specific operations are as follows.

Trust score anomaly detection: If the change Δts of the current round’s trust score deviates from the historical average change $\Delta ts_{avg}$ exceeding the threshold $\sigma_{ts}$, the trust score will be adjusted to

(20) $$\begin{array}{c} ts_{protected}\;=\;ts\;-\;\nu \;\cdot \;\left(\Delta ts\;-\;\Delta ts_{avg}\right) \end{array}$$

where ν controls the adjustment amplitude in order to preserve the overall trend of the trust score while isolating abnormal fluctuations.

Delay anomaly detection: Similarly, if the change in delay Deltadelay deviates from the average value $\Delta delay_{avg}$ by more than $\sigma_{delay}$, the delay will be adjusted to

(21) $$\begin{array}{c} delay_{protected}\;=\;delay\;+\;\nu \;\cdot \;\left(\Delta delay\;-\;\Delta delay_{avg}\right) \end{array}$$

Let $Adv_{oracle}$ denote the probability that an attacker successfully affects the trust score in a round. Assume that the attacker attempts to modify the trust score ts and delay delay in round *t*, and the dynamic protection algorithm in the system performs real-time detection based on the thresholds $\sigma_{ts}$ and $\sigma_{delay}$ and adjustments. The expected value of attack success probability $E(Adv_{oracle})$ satisfies the following inequality:

(22) $$\begin{array}{c} E\left(Adv_{oracle}\right)\;\leq \;\frac{1}{\lambda }\;\cdot \;\left(1\;-\;\frac{\sigma_{ts}}{|\Delta ts\;-\;\Delta ts_{avg}|\;+\;\epsilon }\right)\;\cdot \;\left(1\;-\;\frac{\sigma_{delay}}{|\Delta delay\;-\;\Delta delay_{avg}|\;+\;\epsilon }\right) \end{array}$$

where ϵ is a tiny positive number used to ensure that the denominator is non-zero. It can be seen that as $\sigma_{ts}$ and $\sigma_{delay}$ increase, the attacker’s success probability tends to decrease; that is, the robustness of the protection mechanism increases.

To ensure that the dynamic adjustment of the trust score can restore stability after an attack, we further analyze the expected convergence speed of the adjusted trust score and delay. Assuming that the adjustment coefficient ν is appropriate, the adjusted trust score expectation $E(ts_{protected})$ satisfies the following convergence conditions:

(23) $$\begin{array}{cc} E(ts_{protected}) & \;=\;ts\;+\;\left(\frac{\nu \;\cdot \;\Delta ts_{avg}}{\lambda }\right)\;\cdot \;\left(1\;-\;\frac{\sigma_{ts}}{|\Delta ts\;-\;\Delta ts_{avg}|}\right) \end{array}$$

By controlling the sizes of ν and λ, the dynamic balance of the trust score can be ensured while resisting attacks.

This dynamic protection mechanism monitors changes in trust scores and delays in real time and dynamically adjusts parameters to prevent the impact of malicious manipulation. The above formula analysis shows that the system can effectively maintain the stability of the trust score when it is attacked, thereby enhancing the anti-attack ability and trust reliability of the oracle.

## 5. Results and Discussion

### 5.1. Computational Cost Analysis and Comparison

To demonstrate the efficiency advantages of this mechanism, this section presents an experimental comparison with three existing state-of-the-art schemes, which are the SPChain system proposed by Zou et al. [27], the multi-keyword search inner-product searchable encryption scheme by Liu et al. [28], and the blockchain and cloud–edge-computing-based electronic medical record sharing scheme proposed by Gao et al. [29]. The implementation of the scheme uses Python’s pycrptodome and pypbc modules to construct bilinear pair mappings and multiplication operations on groups and power operations; the hash function uses the SHA3_256 algorithm in the hashlib module; and fingerprinting operations in the cuckoo filter are constructed using the mmh3 non-cryptographic hash algorithm module. The experimental environment is Intel Core i7-9700K processor and 16 GB RAM Ubuntu 20.04 operating system, using the Ethernet test network to simulate the blockchain platform for data interaction, the Blockchain Oracle selected Chainlink. the experiments are chosen to carry out in several respects the storage overhead, the query processing time, and the validation time for comparison and evaluation.

In the initialization phase of the system, storage overhead is an important indicator of system efficiency, and the schemes need to calculate the checksum value of each data, and the initial checksum value calculation is a one-time computational overhead. Therefore, the more data the outsourced dataset contain, the higher the initial computation overhead, the longer the signature construction time, and the more storage space is occupied on the server. The storage overhead that the four verification schemes need to occupy at least under different databases is shown in Table 1.

As can be seen from Table 1, SPChain still requires a large amount of storage space when dealing with large-scale EHR data, although it optimizes the blockchain storage overhead. MK-IPSE improves on storing cryptographic indexes, but the storage requirement rises significantly with the increase in the number of keywords. Gao et al.’s scheme partially relieves the storage pressure through edge computing, but the overall storage overhead is still higher. In contrast, this scheme uses cuckoo filters for checking information storage, which drastically reduces the storage requirement. Specifically, when processing 10,000 records, the storage overhead of this scheme is only 420 KB, while that of SPChain is 19 MB, and that of MK-IPSE is a higher 2.5 MB, compared to which this scheme reduces the storage space requirement by more than 84%. This result shows that this scheme significantly reduces the cost of storage and network communication in large-scale data processing scenarios.

**Figure 4:**
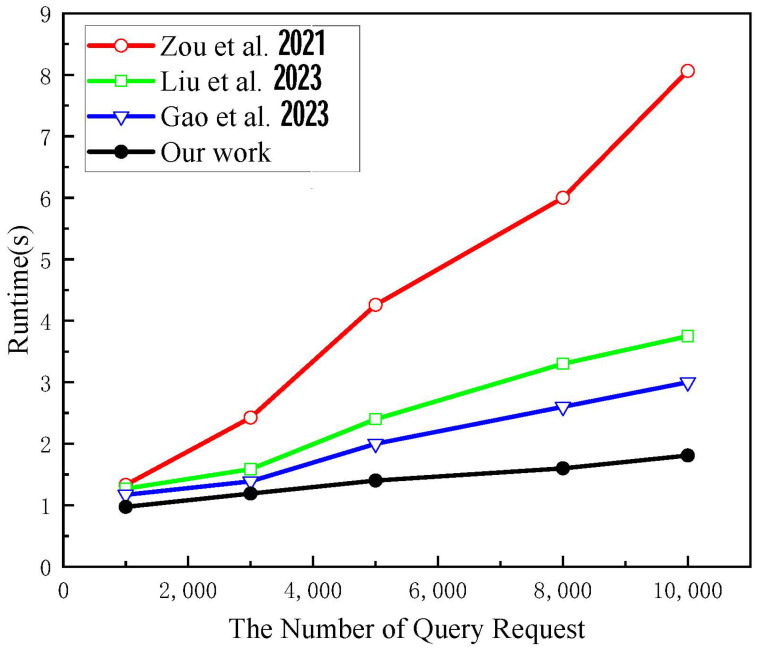
Comparison of retrieval processing time [27,28,29].

The query processing time mainly reflects the response speed of the system to the query request and the construction speed of the verification proof when executing the verification scheme. As shown in Figure 4, the SPChain system has a more substantial query processing time when locating EMR data through specially structured blocks but performs slightly worse when dealing with highly concurrent queries. MK-IPSE responds faster when dealing with multi-keyword queries, but the time overhead increases significantly when the keyword complexity is increased. Gao et al.’s scheme accelerates the query processing through edge computing though. However, the query processing time is still long due to bilinear mapping and power operation. This scheme shows significant advantages in query processing time. When performing 10,000 queries, the average query processing time of this scheme is only 1.8 s, which is about 48% less than MK-IPSE.

**Figure 5:**
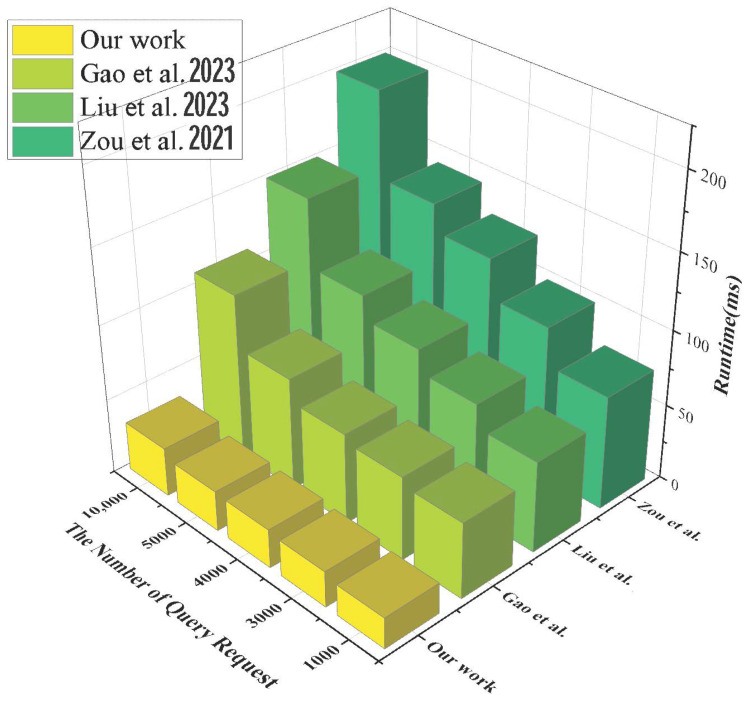
Comparison of retrieval and verification times [27,28,29].

The query verification time is a key indicator of the efficiency of the verification scheme. As shown in Figure 5, the schemes of Zou et al. and Gao et al. have longer verification times due to the complex encryption and re-encryption processes involved. MK-IPSE also has a more significant verification time as it involves multiple keyword-matching operations in the ciphertext search and verification process. In contrast, this scheme case simplifies the verification process by using cuckoo filters, which, with their efficient lookup and matching capabilities, enable this scheme to avoid complex encryption operations when performing verification, thus achieving linear time complexity of the verification process. This means that no matter how the size of the dataset grows, the validation time of this scheme is only linearly related to the number of data entries and is not significantly affected by other factors. Specifically, when dealing with the verification task of 50,000 records, the verification time of the proposed scheme is only 26 ms, which is significantly better than the comparison scheme. Compared with SPChain, the proposed scheme reduces the verification time by about 80%; compared with MK-IPSE, it reduces it by about 50%.

With the above evaluations, the experimental results show that the proposed method exhibits excellent performance in the label construction computation, query processing, and query verification facets. Compared with the existing methods, especially in the verification facet, the performance is outstanding, with high practicability and effectiveness, and can effectively improve the efficiency of data retrieval integrity of the blockchain oracle.

In the comparative analysis with existing solutions, the RIVMD-BO mechanism shows significant performance advantages, but it also has certain implementation limitations:

- Advantages: Compared with other methods, RIVMD-BO performs well in storage and processing performance. Through the efficient storage and fast query capabilities of cuckoo filters, the storage overhead and query verification time are significantly reduced. Especially when processing large-scale medical data, the linear time complexity of this solution ensures good scalability, enabling it to support smart medical scenarios with high concurrency and large amounts of data.
- Disadvantages: When the RIVMD-BO mechanism introduces cuckoo filter technology, it relies on high-quality hash functions and filter parameter settings to ensure a low false positive rate and optimal performance. This mechanism is more sensitive to the selection of filter parameters. If the dataset is frequently updated or the size increases significantly, the filter may face potential problems with an increased false positive rate. In addition, due to the design of the filter, this mechanism still has some room for improvement in terms of real-time processing capabilities compared with multi-keyword matching schemes.

### 5.2. Discussion

In the process of implementing RIVMD-BO, the introduction of the cuckoo filter significantly improved the retrieval efficiency of the system but also brought about potential impacts on scalability and real-time processing capabilities. First of all, the cuckoo filter has high speed and accuracy in data query; especially when the dataset is large, it achieves better scalability by reducing conflicts and lowering the false positive rate. However, in the face of the continuous expansion of dataset size, the cuckoo filter may face the problem of increased hash collisions and rising storage space requirements. This scalability limitation may impact the system’s ability to handle extremely large amounts of medical data. Therefore, to cope with this situation, future system designs can consider dynamic expansion strategies or the architecture of multi-layer filters to mitigate the impact of conflicts on scalability.

In addition, cuckoo filters perform well in real-time data processing and can quickly verify the authenticity and integrity of data items. However, in situations where data need to be updated or deleted frequently, its performance may be degraded, especially in real-time applications where the frequency of data updates is increased. Therefore, in data environments that require a high degree of real-time performance, the cuckoo filter may need to be appropriately optimized, such as by increasing the flexibility of fingerprint calculations or introducing an incremental update mechanism to improve the adaptability of real-time processing.

Overall, the cuckoo filter provides an efficient data integrity verification mechanism for RIVMD-BO while significantly reducing computational overhead. However, there may be a risk of performance degradation in applications with extremely large-scale or high-frequency dynamic updates. Therefore, on the basis of selecting the cuckoo filter, we recommend that the system make trade-offs based on the characteristics of the demand scenario in practical applications, and taking into account the diversity of medical information systems, the cuckoo filter can be further optimized to balance scalability and requirements for real-time data processing capabilities. This approach provides strong support for the interoperability and data management security of smart medical systems while ensuring data verification efficiency.

## 6. Conclusions

This paper proposes a Blockchain Oracle retrieval integrity verification and multi-system data interoperability mechanism for smart healthcare, particularly in the context of the growing prevalence of IoT devices. The primary objective is to maintain the integrity of external data acquired by the smart medical system, thereby enhancing overall security and reliability in an increasingly data-rich environment. To achieve this, we first present a mechanism for real-time integrity verification of data by the Blockchain Oracle. The integration of IoT technology into healthcare systems necessitates efficient verification processes, and we design a verification approach that incorporates cuckoo filter technology, which greatly reduces the computational complexity and increases the efficiency of data verification. Comprehensive security proofs demonstrate that the proposed method is resilient to various attacks and well suited for environments dealing with highly sensitive medical data, particularly as IoT devices contribute vast amounts of data. Moreover, a comparative analysis with existing schemes reveals that our approach is efficient in terms of computational and communication overheads, making it ideal for complex smart healthcare scenarios involving large volumes of data generated by IoT devices.

In the future, we will further optimize the RIVMD-BO mechanism to support a wider range of application scenarios, especially to improve its interoperability between different healthcare systems. In-depth research on the heterogeneity of different medical data systems and data format standardization will help solve key challenges in interoperability and provide a more solid technical foundation for the application of the RIVMD-BO mechanism in a multi-system environment. In addition, we will focus on designing more secure and efficient data verification and message authentication solutions for mobile smart devices in smart medical systems and further study the combination of homomorphic encryption and Blockchain Oracle data authentication mechanisms. By ensuring that the encrypted state of data remains unchanged during the computational process, a higher level of data privacy protection can be achieved to meet the increasingly complex privacy protection needs of smart medical systems. These optimizations will help further improve the overall security and reliability of smart medical systems.

## Figures and Tables

**Table 1:** Comparison of storage overhead.

Number	Zou et al. [27]	Liu et al. [28]	Gao et al. [29]	Our Work (KB)
1000	2300	320	240	45
2000	5100	760	420	85
5000	9000	1650	900	200
10,000	19,000	2500	1600	420

## Data Availability

No new data were created nor analyzed in this study. Data sharing is not applicable to this article.
